# Effects of variation in forest fragment habitat on black howler monkey demography in the unprotected landscape around Palenque National Park, Mexico

**DOI:** 10.7717/peerj.9694

**Published:** 2020-08-10

**Authors:** Keren Klass, Sarie Van Belle, Alvaro Campos-Villanueva, Fernando Mercado Malabet, Alejandro Estrada

**Affiliations:** 1 Department of Anthropology, University of Toronto, Toronto, ON, Canada; 2 Instituto de Biología, Universidad Nacional Autónoma de México, Mexico City, Mexico; 3 Department of Anthropology, University of Texas at Austin, Austin, TX, USA

**Keywords:** *Alouatta pigra*, Demography, Habitat loss and fragmentation, Dispersal, Arboreal primate, Forest fragment habitat quality, Landscape connectivity

## Abstract

Habitat loss and fragmentation are leading threats to biodiversity today, and primates are particularly vulnerable to anthropogenic habitat disturbance. However, few studies have examined how differential effects of variation in forest fragment characteristics on males and females in a primate population may affect demography and population persistence. We quantified the effects of variation in forest fragment characteristics on the within-fragment demography of black howler monkeys (*Alouatta pigra*) in forest fragments around Palenque National Park, Mexico, and how these effects differed between adult males and females. We quantified forest loss in the landscape between 2000 and 2017, and used a redundancy analysis to examine the effects of 15 variables quantifying fragment dimensions, forest composition and physical structure, and isolation on fragment population size and density, the proportion of adult males and females in the fragment population, and the mean number of adult males and females per group in 34 fragments (*N* = 393 monkeys). We hypothesized that (i) population size is positively correlated with fragment area, while population density is negatively correlated, and (ii) the composition of fragment populations results from differential effects of fragment variables on adult males and females. Forest cover decreased by 23.3% from 2000 to 2017. Our results showed a significant effect of fragment variables on population demography in fragments, accounting for 0.69 of the variance in the demographic response variables. Population size increased with fragment area and connectivity, while density decreased. Larger, less isolated fragments with better connectivity, characteristics indicative of abundant secondary growth, and those with more diverse vegetation but lower Simpson’s evenness indices tended to have more adult females per group and a higher proportion of adult females in the population. In contrast, fragments that were largely similar in characteristics of forest composition and structure, but that were more isolated from nearby fragments, had more adult males per group and a higher proportion of adult males. These results may stem from black howler females preferentially remaining in natal groups and fragments when possible, and dispersing shorter distances when they disperse, while males may be more likely to disperse between fragments, traveling longer distances through the matrix to more isolated fragments. These differential effects on males and females have important conservation implications: if females are more abundant in larger, less isolated fragments, while males are more abundant in more isolated fragments, then to effectively conserve this population, both landscape connectivity and fragment areas should be maintained and increased.

## Introduction

Anthropogenic habitat fragmentation is a landscape-scale process that involves both habitat loss and the breaking apart of habitat. As it progresses, fragmentation leads to an increased number of habitat patches in the landscape, a decrease in patch sizes, an increase in their isolation, and a reduction in total habitat amount in the landscape (Fahrig et al., 2019). Habitat loss and fragmentation are leading threats to biodiversity today (Haddad et al., 2015), and given the scale and severity of these threats, it is imperative to understand the myriad ways they affect species, to better mitigate their impacts.

In primates, a taxon that is particularly vulnerable to anthropogenic habitat disturbance (De Almeida-Rocha, Peres & Oliveira, 2017), few studies have examined the differential effects of multiple fragmented landscape characteristics on males and females, and subsequent changes to population demography and social organization. Here, our main objective was to quantify the effects of variation in forest fragment characteristics on the within-fragment population size, density and composition of black howler monkeys (*Alouatta pigra*) in unprotected forest fragments around Palenque National Park (PNP), Chiapas, Mexico, and specifically, to examine whether variation in forest fragment habitat had different effects on adult males and females.

In fragmented landscapes, animal populations are often simultaneously affected by: (1) characteristics of the fragments they inhabit (e.g., fragment area); and (2) connectivity and habitat amount around the fragment and at the landscape scale (Arroyo-Rodriguez et al., 2013a; Heinrichs et al., 2015; Fletcher et al., 2018). Landscape connectivity is largely determined by the matrix, the altered land cover types surrounding habitat fragments (Eycott et al., 2012).

At the local scale, characteristics of fragments themselves may shape animal populations living in them in various ways. In primates, smaller areas often result in higher population densities (Irwin, 2008), while limiting the number and size of groups and thus overall population size (Gilbert, 2003; Ewers & Didham, 2006) and local mate availability. Increased density in animal populations may lead to more resource competition (Bowler & Benton, 2005) or altered social dynamics (Boudjemadi, Lecomte & Clobert, 1999), which may also affect dispersal rates (Bowler & Benton, 2005) and demography (Richard et al., 2002).

Habitat quality is often correlated with fragment size, shape, and isolation (Arroyo-Rodriguez & Mandujano, 2006). As area decreases and isolation and fragment shape complexity increase, the composition and diversity of vegetation may be increasingly modified relative to primary, undisturbed rainforest, with larger amounts of edge-affected habitat, that can negatively affect the quality, quantity and variety of resources within the fragment (Anzurres-Dadda & Manson, 2007; Ewers & Didham, 2007; Arroyo-Rodriguez & Dias, 2010; Laurance et al., 2017). Changing resource availability can affect the carrying capacity of fragments, and the population sizes of species in those fragments (Arroyo-Rodriguez et al., 2007). Beyond the tree species composition in forest fragments, primates may also be sensitive to vegetation structure; for example, for canopy-dwelling species, tree height may be an important factor in determining habitat suitability (Anzurres-Dadda & Manson, 2007). In forest fragments, altered environmental variables (e.g., light exposure) and an increase in edge effects can lead to changes in the physical structure (e.g., tree height and density) of the forest (Arroyo-Rodriguez & Dias, 2010; Puig-Lagunes et al., 2016), further reducing the suitability of habitat for some arboreal primates.

For arboreal species, the costs (e.g., energy expenditure; Bowler & Benton, 2005) of moving in the matrix may be high, as they depend on forest for food, refuge, sleeping and reproductive sites, and are adapted for arboreal movement (Lancaster et al., 2011; Pozo-Montuy, Serio-Silva & Bonilla-Sanchez, 2011). These costs are shaped by matrix composition, fragment isolation, the amount of habitat remaining in the landscape around the fragment, and the presence of stepping stones or treelines in the matrix (Bender, Tischendorf & Fahrig, 2003; Prugh et al., 2008; Arroyo-Rodriguez & Dias, 2010; Pozo-Montuy, Serio-Silva & Bonilla-Sanchez, 2011).

High costs of movement through the matrix may affect the demographics of animal populations in fragments, for example by increasing mortality rates among individuals dispersing between habitat patches (e.g., Iberian lynx, *Felis pardina*, Ferreras et al., 1992; root vole, *Micrototus oeconomus*, Aars, Johannesen & Ims, 1999). The challenges of moving through the matrix may also affect the cost-benefit balance of individuals’ decisions whether to stay in their current patch or leave to find new resources or reproductive opportunities (Baguette et al., 2012), potentially leading to increased philopatry, with attendant changes to group composition and genetics (e.g., black-and-gold howler monkeys, *Alouatta caraya*, Oklander & Corach, 2013).

In a fragmented landscape, both resources (Heinrichs et al., 2015; Rangel-Negrín et al., 2018) and mating opportunities (Gros, Poethke & Hovestadt, 2009) may be subject to substantial spatiotemporal variation. Owing to differences in reproductive costs and investments (Trivers, 1972), ecological theory predicts that female mammals will often compete more over food resources, while males compete more over reproductive opportunities (Gros, Poethke & Hovestadt, 2009). Thus, we might expect males and females to be affected by fragment and landscape characteristics and variation in resource availability in different ways, further altering the demographic composition of populations in habitat fragments (Amos et al., 2014).

Reduced resource availability may affect the sex ratio of dispersers; for example, in a population of mantled howler monkeys (*Alouatta palliata*), fragmentation resulted in increased dispersal rates in females, potentially driven by a reduction in resource availability (Jones, 2005). Higher mortality rates during dispersal in species with sex-biased dispersal may alter sex ratios in fragments, with the dispersing sex becoming rarer (e.g., male-biased sex ratios in small, isolated bird populations with female dispersal, Dale, 2001; female-biased sex ratios in agile antechinus, *Antechinus agilis*, with male-biased dispersal in forest fragments, Banks et al., 2005). This process was proposed as an explanation for an adult sex ratio skewed towards females in black-and-gold howler monkeys in fragments (Zunino et al., 2007).

Evidence suggests that moving individuals are more likely to encounter fragments with more complex shapes and more edges (Ewers & Didham, 2006), which may increase dispersal rates into fragments with high shape indices; if one sex becomes more philopatric in forest fragments, then fragment shape may have a greater effect on the dispersing sex. In landscapes that limit movement, if fragments are located near a large source population (mainland-island model of metapopulation dynamics; Hanski, 1998) of a species with sex-biased dispersal, one might find more individuals of the dispersing sex in fragments closer to the source population.

Black howlers are Endangered (Marsh et al., 2008), arboreal primates endemic to tropical rain forests in Mexico, Belize, and Guatemala. Black howlers are social animals, living in groups that range from 2 to 16 individuals with a variable group structure (multi-or single male and multi- or single-female; Van Belle & Estrada, 2006). They exhibit mixed mating strategies (females copulating with one resident male or several, or extra-group males; Van Belle & Bicca-Marques, 2015) and flexible dispersal behavior in both sexes, with both males and females sometimes remaining in the natal group, although evidence suggests that dispersal is male-biased (Van Belle et al., 2012). When females disperse, it occasionally results from targeted aggression from older resident females (Dias et al., 2015). Males may immigrate into new groups solitarily or in coalitions of related males (Van Belle et al., 2012).

Black howlers are generally considered adaptable to habitat fragmentation. Groups are found in very small forest fragments (<2 ha; Rangel-Negrín et al., 2018; Klass, Van Belle & Estrada, 2020), and black howlers display considerable flexibility in their folivorous/frugivorous diet, which may allow them to persist in degraded habitat (Dias et al., 2014; Bicca-Marques, Chaves & Pacheco Hass, 2020). However, they prefer tall trees (>15 m in height; Gonzalez-Kirchner, 1998), which may be rarer in small or secondary growth fragments. Moreover, studies have shown that residing in poorer quality habitat or smaller fragments can have negative impacts on their health and fitness (Rangel-Negrín et al., 2018), and that some black howler demographic parameters (population growth, infant survival) are adversely affected by decreasing fragment sizes and increased habitat disturbance (Rangel-Negrín et al., 2018).

The composition and high per-fragment density of our study population have been largely stable for ~20 years, but it has higher densities, and fewer adult males and fewer multi-male groups, than the adjacent protected, continuous population in PNP (Klass, Van Belle & Estrada, 2020). In contrast, the proportion of adult females in the population, the average number of adult females per group, and the proportion of multi-female groups are similar in both populations. These differences indicate that black howler males and females are affected by habitat loss and fragmentation in different ways. If this is the case, we would expect to see differential effects of fragment characteristics on the sexes expressed in the within-fragment demography (Amos et al., 2014). In this study, we examined how variation in different characteristics of (i) the fragments themselves, including fragment dimensions, forest structure and forest composition, and (ii) characteristics of the landscape surrounding each fragment that are indicative of connectivity, affect the size, density, and composition of the population of black howlers within forest fragments. We tested the following hypotheses:

1. Characteristics of forest fragments and landscape connectivity in the vicinity of fragments have a measurable, significant effect on the population size and density of black howlers living in fragments. Specifically, we predict that population size is positively correlated with fragment area, and population density is negatively correlated with area.
2. Variation in the composition of black howler populations in fragments is shaped by differential effects of fragment characteristics and fragment connectivity on adult males and females. Specifically, we predict that:

- The proportion of adult females in the fragment and the mean number of adult females/group are largely shaped by fragment variables (e.g., fragment area, stem density), with positive correlations with variables indicative of good habitat quality (i.e., habitat that is more similar to primary, undisturbed rainforest), as adult females compete more over food resources and territory within fragments (Gros, Poethke & Hovestadt, 2009) and thus tend to become more philopatric in fragmented landscapes.
- The proportion of adult males in the fragment and mean number of adult males/group in forest fragments are shaped largely by connectivity variables (e.g., distance to nearest fragment), as well as the shape index of fragments, with positive correlations with variables indicative of greater connectivity, because adult males disperse between fragments at higher rates than females and experience high dispersal costs in the matrix.

Lastly, while there is evidence of deforestation in the study area up to 2001 (Estrada et al., 2002), we also aimed to determine whether and where forest loss was currently ongoing in the study landscape.

## Materials and Methods

### Study site

**Figure 1:**
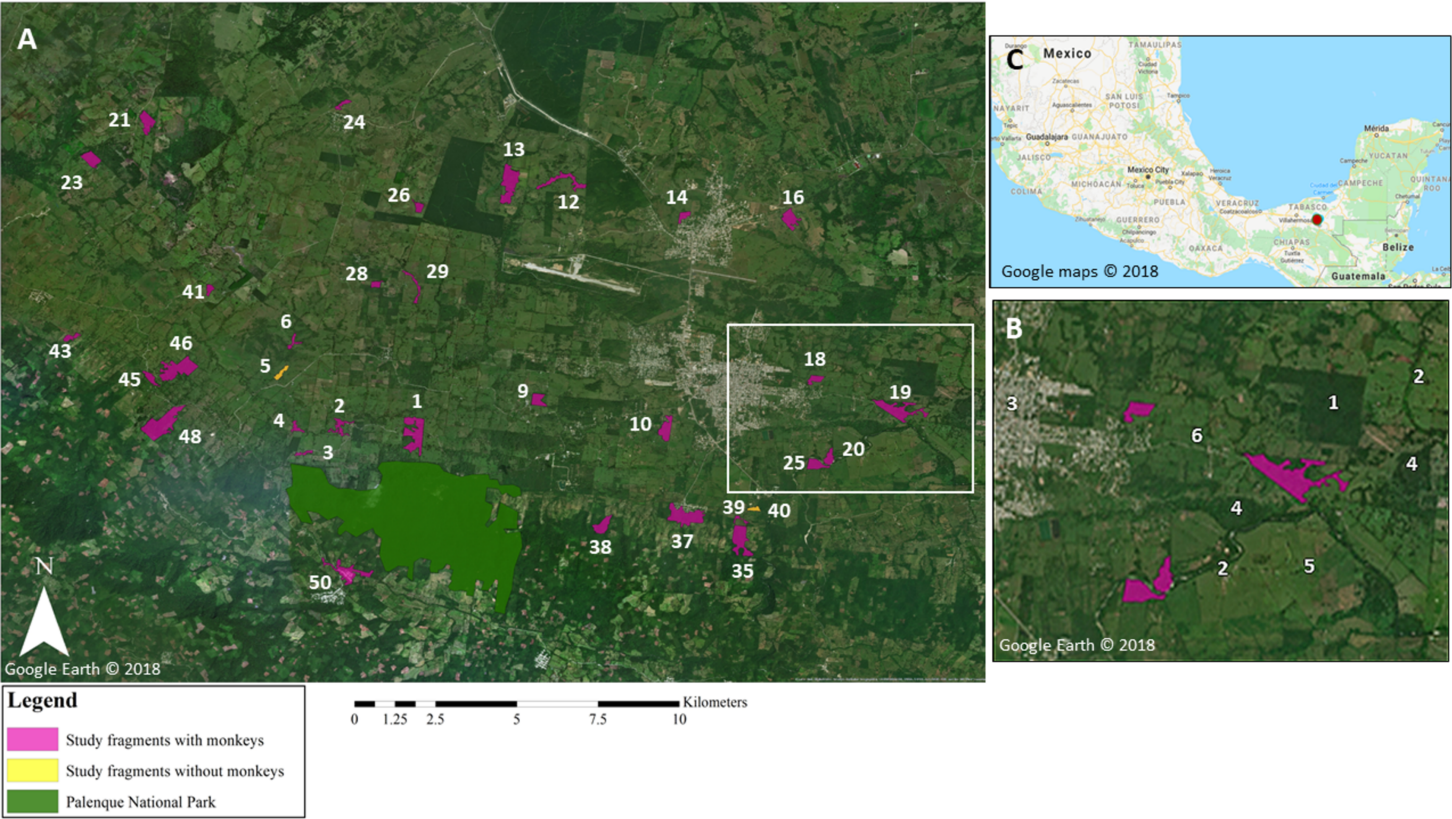
Map of study area. (A) Map highlighting the core area of Palenque National Park (PNP), and the 34 study fragments located within 10 km of PNP. In white: fragment ID numbers; one fragment is not visible on the map due to its small size (fragment 49; 0.2 ha). White rectangle denotes area shown in greater detail in (B). Google Earth© 2018. (B) Enlarged section of study area highlighting different elements in the landscape: (1) oil palm plantation; (2) riparian corridors; (3) town; (4) forest fragments not included in this study; (5) pastureland matrix; (6) road. Google Earth© 2018. (C) Location of study site within Chiapas, Mexico, marked by red dot. Google Maps© 2018. ****

Our study was conducted in 34 forest fragments located within a ~10 km radius of PNP, Mexico (17°28′N, 92°03′W; Fig. 1). PNP encompasses 1,771 ha, ~1,000 ha of which are primary and secondary tall evergreen and tropical rainforest. PNP contains a stable population of numerous black howler groups (Van Belle et al., 2012; Klass, Van Belle & Estrada, 2020). The study fragments (Table 1; Table SM1) ranged in size from 0.2 to 36.2 ha (mean = 11.1 ha ± 9.8 SD; median size = 7.5 ha), for a total area of 377 ha. The fragments ranged in elevation from 34 to 380 m and were located 0.3–9.7 km (mean = 5.3 km ± 2.7) from the core area of PNP.

**Table 1:** Summary of explanatory and response variable values for all study fragments.

	Variable	Mean (SD)	Range
Explanatory (fragment) variables	Area (ha)	11.1 (9.8)	0.2–36.2
	Shape index	2.2 (0.7)	1.5–4.1
	Stem density (stems/1,000 m^2^)^a^	0.04 (0.02)	0.01–0.08
	Min. tree DBH (cm)^a^	10.7 (1.4)	10.0–15.3
	Max. tree DBH (cm)^a^	112.1 (72.8)	42.2–350.0
	Min. tree height (cm)^a^	3.6 (2.3)	1.0–13.5
	Max. tree height (cm)^a^	25.9 (6.4)	12.8–43.0
	Shannon’s diversity index (tree genera)^a^	2.2 (0.8)	0.0–3.2
	Simpson’s evenness index (tree genera)^a^	0.8 (0.6)	0.2–3.0
	No. of stumps^a^	4.3 (3.4)	0–12
	Mean stump DBH (cm)^a^	27.2 (10.4)	13.7–64.3
	Isolation distance to nearest fragment (m)	184.6 (177.7)	8–549
	Isolation distance to PNP (m)	5,312.3 (2,727.2)	335–9,671
	Number of treelines extending from fragment	2.6 (2.1)	0–8
	Proportion forest cover in buffer around fragment	0.27 (0.15)	0.1–0.7
Response (demographic) variables	Total no. of individuals per fragment	11.6 (8.7)	0–38
	Fragment population density (ind/ha)^b^	2.6 (7.2)	0.3–42.1
	Proportion of AM in the fragment population	0.2 (0.07)	0.10–0.3
	Proportion of AF in the fragment population	0.3 (0.10)	0.20–0.8
	Mean fragment group size	6.4 (2.2)	3–11
	Mean #AM/group in the fragment	1.2 (0.5)	0–3
	Mean #AF/group in the fragment	2.2 (0.7)	1–4

**Notes:**

a Variables that were calculated for 32/34 forest fragments.

b Excluding the smallest fragment of 0.2 ha, which had one group of eight monkeys, mean density per fragment was 1.4 ± 0.8 (range: 0.3–3.4 ind/ha).

**Figure 2:**
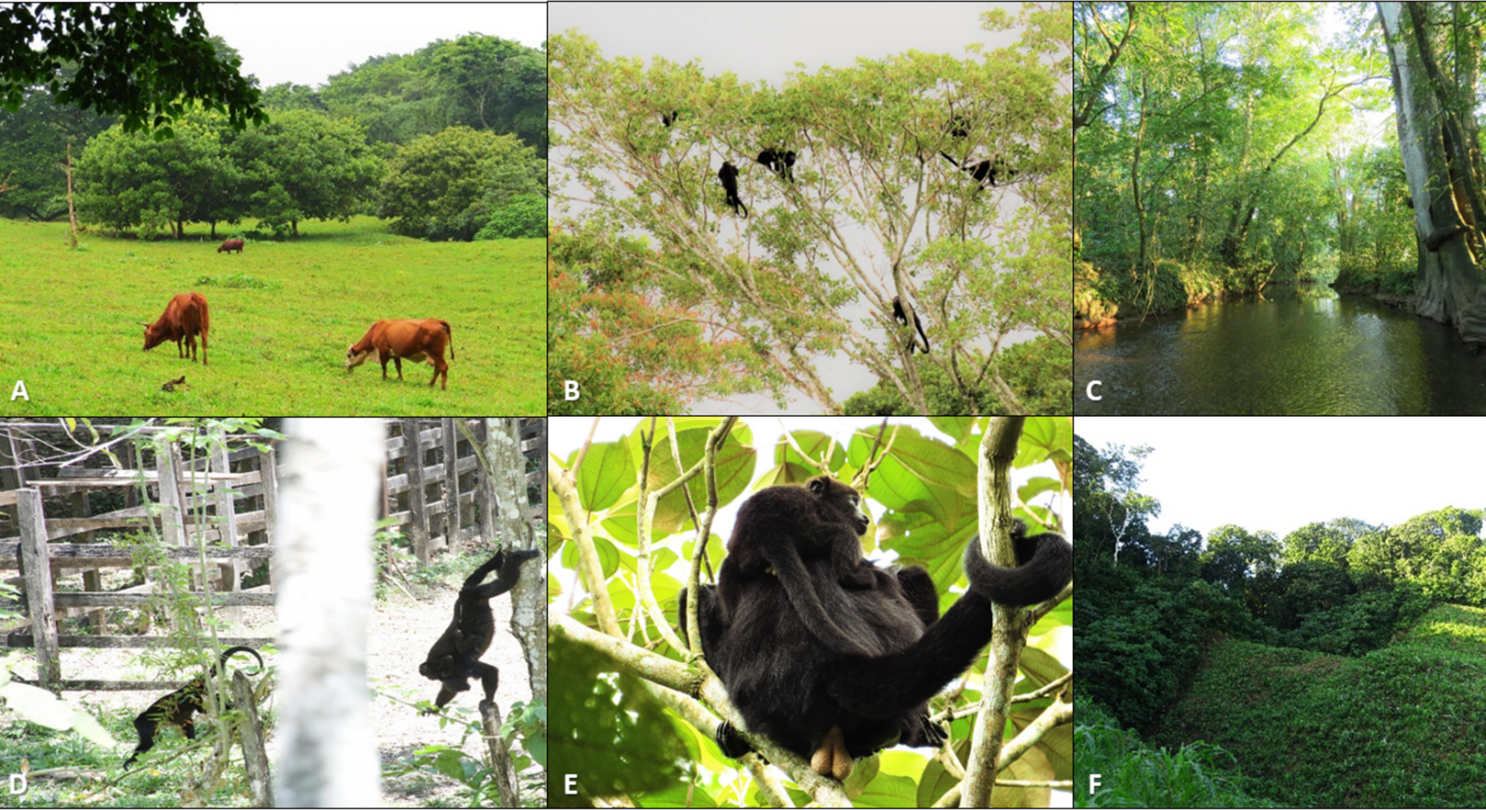
Photos detailing characteristics of the study fragments and their black howler monkey populations. (A) ****Cattle grazing in typical pastureland matrix found around study fragments (photo credit: Keren Klass); (B) a group of black howlers in a mature, tall tree at the edge of a forest fragment (photo credit: Keren Klass); (C) stream running through an area of mainly secondary growth in a forest fragment, with a single extremely large, mature *Ficus insipida* tree on the right (photo credit: Keren Klass); (D) black howler monkeys using low barbed wire fencing to cross the matrix between two stands of trees (photo credit: Alvaro Campos Villanueva); (E) adult male carrying an infant in a *Miconia argentea* tree, a secondary growth species commonly found in the study fragments (photo credit: Keren Klass); (F) a forest fragment bordered by low secondary growth ****and cornfield matrix (photo credit: Keren Klass).

Fragments around PNP were found in pastureland matrix and on steep slopes (Fig. 2). Many streams formed the basis of narrow riparian corridors of remnant primary forest (Fig. 2C). Fragments were mainly composed of secondary growth forest and remnant primary forest, and were subject to disturbance (e.g., cattle grazing) of varying intensity. Human activities in the matrix consisted mainly of cattle pastures and agriculture (Figs. 1 and 2), including oil palm plantations; some fragments were adjacent to urban areas. Interviews of local people indicated that anthropogenic deforestation and fragmentation began about a century ago (A. Estrada, 2015, unpublished data). Between 1984 and 2001 ~33% of forest cover was lost in and around PNP and the number of fragments increased (Estrada et al., 2002).

### Data collection

#### Population composition (response) variables

Data collection was conducted under a research permit from Dirección General de Vida Silvestre de la SEMARNAT (SGPA/DGVS/05700/17; SGPA/DGVS/002486/18) and ethical approval from the University of Toronto (AUP 20011957). All applicable international, national, and/or institutional guidelines for the care and use of animals were followed in this research.

The study population included all black howlers observed between September 2017 and May 2018 in the study fragments. Monkeys were observed in 32 of the 34 study fragments (mean number of days per fragment: 3.8; range: 1–7 days; Fig. 1). We observed 60 groups and eight solitary individuals (*N* = 393). We observed groups for an average of 10.9 ± 6.8 h (range = 1.1–38.9 h; average time spent with solitary individuals = 4.0 ± 3.5 h, range = 0.2–8.2 h; for further details on the collection of demographic data, see Klass, Van Belle & Estrada, 2020).

The number of monkeys per fragment ranged from 0 to 38 (mean = 11.6 ± 8.7 ind) and the number of groups per fragment ranged from 0 to 6 (mean = 1.8 ± 1.4). The population was composed of 53% adults (*N* = 208), 33% juveniles (*N* = 131), and 14% infants (*N* = 54). Of the total population, 19% (*N* = 75) were adult males (AM) and 34% (*N* = 133) adult females (AF). The adult sex ratio (M:F) was 0.56:1, and the infant sex ratio was 1:1. Twenty-one percent of groups were multimale and 82% were multifemale (Table SM1; Klass, Van Belle & Estrada, 2020).

To analyze the effect of variation in fragment habitat on black howler demography, we quantified population size, density, and composition, and average group composition, for each study fragment. Specifically, we calculated the following seven variables: total number of individuals in the fragment (including solitary individuals), fragment population density, the proportions of AM and AF of the total fragment population, average group size in the fragment, and the average number of AM and AF per group in the fragment (Table 1).

### Fragment (explanatory) variables

We analyzed the effect of fragment-scale variables (hereafter referred to as fragment or explanatory variables; Tables 1 and 2) of different types, including dimensions, forest physical structure, and forest composition, and variables quantifying landscape connectivity in the vicinity (10–1,000s of m) of each fragment. We collected data on fragment area, shape, isolation from nearest fragment and from PNP, and number of treelines extending from the fragment for all 34 study fragments, by examination of high-resolution (1 m^2^) satellite images of the study area in Google Earth© (2018), combined with on-the-ground validation using handheld GPS data during surveys of each fragment and its perimeter (Tables 1 and 2).

**Table 2:** Method of calculation, description, and variance inflation factor (VIF) scores for all fragment (explanatory) variables.

Fragment variables	Method of calculation	Description	VIF score
Area (ha)	Area tool in Google Earth©	Fragment size may positively correlate with fragment quality: fragmentation alters tree species composition in fragments, and a greater proportion of smaller fragments is affected by edge effects	2.3
Shape index	Shape index (SI) = fragment perimeter/(√(fragment area) × π)	SI = 1 indicates a perfect circle; the higher the index, the more irregular the shape (Arroyo-Rodriguez et al., 2013b). SI negatively correlates with habitat quality for edge-averse species (Ewers & Didham, 2007), but complex shapes may contribute to connectivity (Ewers & Didham, 2006)	1.9
Stem density	Stem density = (number of trees sampled of DBH ≥ 10 cm)/(area sampled in transects (1,000 m^2^))	In mature, primary forest, lower stem density is expected, because there more large, mature trees and fewer trees per given area. In secondary/new growth forest, in forest with more disturbance, or forest with more new edge habitat, stem density would be higher (Wright, 2005; Santos et al., 2008)	3.4
Minimum tree DBH	Smallest diameter of tree (DBH ≥ 10 cm) sampled in fragment transects	Indicative of age/maturity of the forest or successional stage	2.3
Maximum tree DBH	Largest diameter of tree (of DBH ≥ 10 cm) sampled in fragment transects	Indicative of age/maturity of the forest	3.0
Minimum tree height	Smallest height of tree (DBH ≥ 10 cm) found in fragment transects	Indicative of age/maturity of the forest or successional stage. *Alouatta* spp. are also known to prefer tall trees (Gonzalez-Kirchner, 1998)	2.8
Maximum tree height	Largest height of tree (DBH ≥ 10 cm) found in fragment transects		2.1
Number of stumps	Number of stumps (DBH ≥ 10 cm) sampled in transects	Indicative of natural or human disturbance (e.g., tree mortality/logging; McClennan & Plumptre, 2012)	3.2
Mean DBH of stumps	Mean DBH of stumps (DBH ≥ 10 cm) sampled in fragment transects	Indicates whether dead trees in the fragment were mature, large trees, or young, small trees	1.7
Shannon Index H′ (genera)	${\rm H}^{\prime} = \sum_{i=1}^{S}-({\rm Pi} * {\rm In\ Pi})$ Pi = fraction of population made up of type *i*; *S* = total numbers of types	A quantitative measure that reflects how many different genera there are in the dataset, and simultaneously considers how evenly individual trees are distributed among those types. A higher H′ indicates a highly diverse (and evenly distributed) dataset (Morris et al., 2014)	5.5
Simpson’s Evenness *E* (genera)	*E* = (1/ΣPi^2^)/*S* Pi = the proportion of individuals belonging to type *i*; *S* = total number of types	Indicative of the degree to which a fragment is dominated by a single or a few genera, with high values indicating that relatively equal numbers of individual trees belong to each genus (Morris et al., 2014)	4.2
Distance to the nearest fragment	Distance (m) to nearest fragment >1 ha in size, regardless of fragment occupancy. Measured with the distance tool in Google Earth©	Measures how close the monkeys in fragment *X* are to another patch of forest.	2.6
Distance to PNP	Distance (m) to edge of core area of PNP. Measured with the distance tool in Google Earth©	Measures how close the monkeys in fragment *X* are to a potential “source population” in a relatively large, undisturbed forest (Pulliam, 1988)	2.4
Number of tree lines extending from fragment	Count of number of tree lines (e.g., living fences) of any length extending from fragment	Measure of the difficulty for monkeys to leave or enter the fragment, and their ability to minimize time forced to walk on the ground while traversing the matrix	2.4
Proportion of forest cover in the buffer	Proportion of forest within a pre-defined buffer zone around a fragment; buffer radius = dispersal distance = 7 × (diameter of home range size^a^) (Bowman et al., 2002)	An area-based isolation metric; this alternative measure of isolation may be more appropriate for arboreal species that are able, but reluctant, to travel terrestrially (Arroyo-Rodriguez & Dias, 2010)	3.9

**Note:**

a In this formula, we used the diameter of the mean home range size of five groups in PNP: Motiepa group (7 ha), Pakal group (10.8 ha), Naha group (16.1 ha), Unites group (8.6 ha), Balam group in 2016 (6.5 ha), Balam group in 2017 (6.7 ha). Mean home range size = 9.3 ha.

We calculated an area-based isolation measure (Bender, Tischendorf & Fahrig, 2003), the proportion of forest habitat present within a pre-defined buffer zone around each fragment, excluding the area of the fragment itself. The size of the buffer was equivalent to a circle with a radius of the study species’ median dispersal distance. When dispersal distance has not been directly measured, it can be estimated based on the species’ home range size (Bowman et al., 2002). We used the mean home range size (9.3 ha) of five groups studied long term in PNP (Table 2), in the following formula (Bowman et al., 2002):

(a) dispersal distance = 7 × (diameter of home range size)

which gave an estimated median dispersal distance, and buffer radius, of 2,400 m.

In ArcGIS (ESRI, 2018), we calculated the proportion of forest cover in 2017 using the University of Maryland’s open access Global Forest Change Dataset (Hansen et al., 2013). We defined forest as pixels (30 m^2^) with ≥70% canopy cover. To determine this cut-off point, we first calculated the range and distribution of canopy cover values of random points in the study fragments and PNP at two time-steps, 2000 and 2017, to identify the canopy cover values of known howler monkey habitat. The first quartile value in the distribution of % canopy cover represents 25% of the data; in other words, 75% of the pixels sampled from the study fragments and PNP had canopy cover values greater than the first quartile value. In 2000, the first quartile was 73% canopy cover, and in 2017 it was 67.75%. We used the average of these values, 70% canopy cover, as the minimum to define pixels as “forest”; all other pixels were defined as “matrix”. We then calculated the proportion of forest in the buffer area around each fragment.

We collected data on stem density, tree genus (from which we calculated the Shannon’s diversity and Simpson’s evenness indices; Tables 1 and 2), tree height and diameter at breast height (DBH), and the number and DBH of stumps in 32 of 34 fragments using a modified Gentry Protocol (Gentry, 1982; Dias et al., 2014). Within each fragment, we recorded tree genus identity and DBH for all trees with DBH ≥ 10 cm along 10 transects, 50 m long and 2 m wide (total transect area per fragment = 1,000 m^2^). We recorded tree height for all trees with DBH ≥ 10 cm using a laser rangefinder (Larjavaara & Muller-Landau, 2013), and noted the DBH of all stumps with DBH ≥ 10 cm. Most trees were identified to the level of genus; the proportion of sampled trees per fragment which we were unable to identify ranged from 0 to 0.44 (mean = 0.14 ± 0.12). A total of 98 genera from 38 families were identified in the study fragments (mean = 16 ± 8.04 genera/fragment, range = 1–30; see Table SM2 for full list of genera).

### Statistical analyses

#### Forest cover change and landscape connectivity

In ArcGIS, we calculated forest cover (see “Fragment (explanatory) variables” for details on the calculation of “forest” vs. “matrix”) for an area of 926 km^2^ (92,605 ha) around PNP for two time steps, 2000 and 2017, using the Hansen et al. (2013) dataset. We subtracted the area of forest cover found in 2017 from that found in 2000, to arrive at the amount of forest cover lost in that 17-year period, and the remaining proportion of the landscape with forest cover in 2017.

We ran the 2017 forest cover layer through FRAGSTATS (McGarigal, Cushman & Ene, 2012) in R using the *ConnCompLabel()* and *PatchStat()* functions in the SDMTools (ver. 1.1–221) package (VanDerWal et al., 2014), to determine whether some fragments clustered together with greater connectivity in the landscape. The program used an eight-neighbor rule and returned a total of >6,000 fragments in the study area, using the 30 m^2^ pixel size and definition of forest = >70% canopy cover of the input layer.

**Figure 3:**
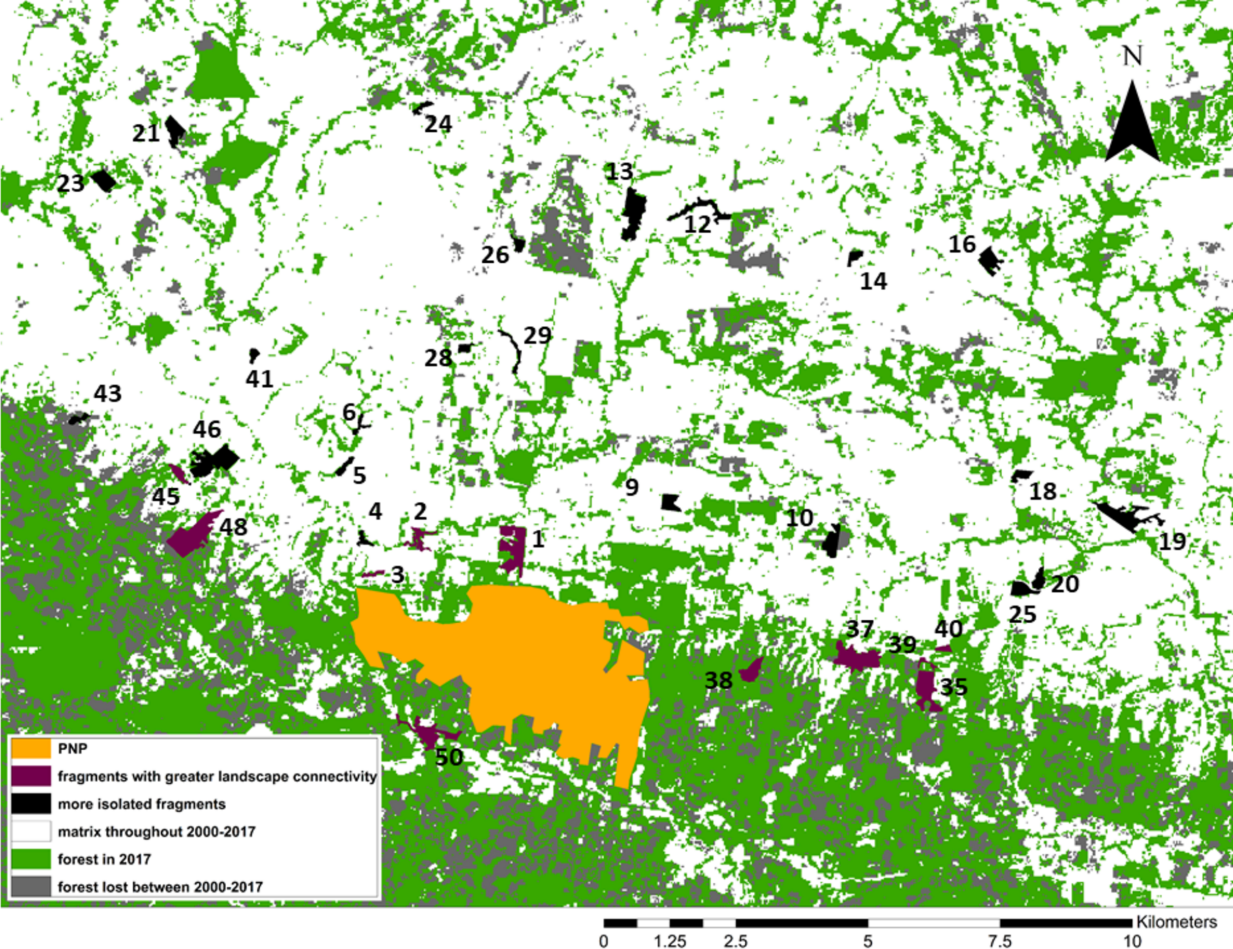
Forest loss and landscape connectivity in the study area. Fragment ID numbers noted in black; one fragment (#49) not visible on the map due to its small size.

We identified our study fragments among the >6,000 defined by FRAGSTATS and found that 11/34 were embedded in what the program identified as one very large patch of forest including PNP and extending to the east and west of the park (Fig. 3). Although higher-resolution Google Earth (2018©) images and on-the-ground validation identified these fragments as individual patches of forest, these results indicated greater connectivity among these fragments, compared to the remaining 23. We used one-tailed Mann–Whitney *U* tests to compare the fragment variables between the two sets of fragments (11 “connected” and 23 “unconnected”; Fig. 3).

#### Redundancy analysis

We first explored the individual relationships among our explanatory and response variables by creating a Spearman correlation heatmap of all variables, using the *rcorr*() function in the Hmisc package in R (Harrell et al., 2016) and the *corrplot*() function in the corrplot package (Wei & Simko, 2017).

We then used redundancy analysis (RDA) to quantify the proportion of the variance in the demographic variables that was explained by the fragment variables and investigate which fragment variables affected different aspects of black howler population demography in forest fragments. Redundancy analysis is a linear ordination method combining Principal Component Analysis (PCA) with multiple regression, which allows one to simultaneously test the effects of a dataset of multiple explanatory variables on a dataset of multiple response variables (Legendre, Oksanen & Ter Braak, 2011). RDA computes orthogonal axes that are the linear combinations of all explanatory variables that best explain the variation in the response variables. RDA is a constrained ordination procedure, meaning that in this test, unlike in PCA alone, the axes explain the variation in the response variable matrix.

The overall variance in the response variables is partitioned into constrained and unconstrained fractions, where the constrained fraction is the amount of variance explained by the explanatory variables. This amount is expressed as a proportion and is equivalent to the *R*^2^ value in multiple regression. Like the unadjusted *R*^2^ of a multiple regression analysis, this *R*^2^ can be upwardly biased when testing large numbers of explanatory variables (Peres-Neto et al., 2006). One can compute an adjusted *R*^2^ value, which Borcard, Gillet & Legendre (2018) recommended doing when the number of explanatory variables is greater than half the sample size. Given that this was not the case in our analysis, we presented the unadjusted *R*^2^ value in our results.

We conducted RDA analyses in R using the vegan 2.5–6 package (Oksanen et al., 2016). Due to their different measurement scales, we standardized the response data by using the option scale = TRUE in vegan’s *rda*() function. We transformed the explanatory data by centering the variables (subtracting the variable’s mean from each value). We tested the global null hypothesis of no linear relationships between explanatory and response variables, the statistical significance of each RDA axis, and the statistical significance of the marginal effect of each explanatory variable in the model with permutation tests (1,000 permutations) in vegan (Legendre, Oksanen & Ter Braak, 2011; Oksanen et al., 2016).

Results are displayed in a triplot using scaling 2. In this scaling, the angles between response and explanatory variables, and among pairs of response or explanatory variables, reflect their linear correlations, which are equal to the cosine of the angle between vectors. For example, an angle of 90° represents two variables that are uncorrelated, as cos(90) = 0, and an angle of 180° represents two variables that are strongly negatively correlated (Van Eeuwijk, 1992). The strength of a variable’s contribution to the ordination axes is expressed by the length of an explanatory variable’s arrow (Ter Braak, 1994), and numerically by variables’ triplot scores.

To determine the degree of collinearity among all explanatory variables we examined their variance inflation factors (VIFs) with the *vif.cca*() function in the vegan package (Oksanen et al., 2016). A high VIF (>20) indicates strong collinearity, although there is some disagreement in the literature regarding the “correct” VIF score cut-off point for inclusion in an RDA model (e.g., 2, 4, 10, 20; O’Brien, 2007; Vu, Muttaqi & Agalgaonkar, 2015). We examined the VIF scores of our full set of 15 explanatory variables, and found that all were <6 (Table 2), which is considered a relatively conservative cut-off point (O’Brien, 2007; Borcard, Gillet & Legendre, 2018). We therefore retained all explanatory variables, 11 of which were characteristics of the fragments themselves (e.g., size, shape, tree stem density) and four that quantified the isolation of the fragments in the landscape (e.g., distance to nearest fragment).

## Results

### Forest loss and connectivity

From 2000 to 2017, forest cover in the study area decreased from 46,395 ha (50% forested) to 35,582 ha to (38.4% forested). Put differently, 23.3% of the forest cover present in 2000 was lost by 2017 (Fig. 3). Our study area is clearly composed of a northern section in which most forest cover had already been cleared by 2000, and a southern section, extending to the east, west, and south of PNP, where larger swathes of forest cover remain. While this area still contains more forest than the northern section, the forest loss analysis shows that most of the deforestation between 2000 and 2017 occurred in this southern area, making this previously large and relatively intact stretch of forest smaller and increasingly fragmented (Fig. 3).

The 11 fragments FRAGSTATS identified as more connected are located within or adjacent to this southern area. When comparing them to the remaining 23 fragments (Fig. 3), we found that 5/15 fragment variables differed significantly between the two groups: maximum tree height was significantly larger in the connected group (Mann–Whitney Tests: *U* = 48, *z* = −2.50, *p* = 0.0062); Simpson’s evenness index was significantly higher in the connected group (*U* = 51, *z* = −2.38, *p* = 0.0087); Shannon’s index was significantly higher in the connected group (*U* = 28.5, *z* = −3.29, *p* = 0.001); distance to PNP was significantly shorter in the connected group (*U* = 50, *z* = 2.80, *p* = 0.0026); and the proportion of forest cover in the fragment buffer areas was significantly larger in the connected group (*U* = 15, *z* = −4.09, *p* < 0.00001).

### Effects of fragment variables on black howler population size, density and composition

**Figure 4:**
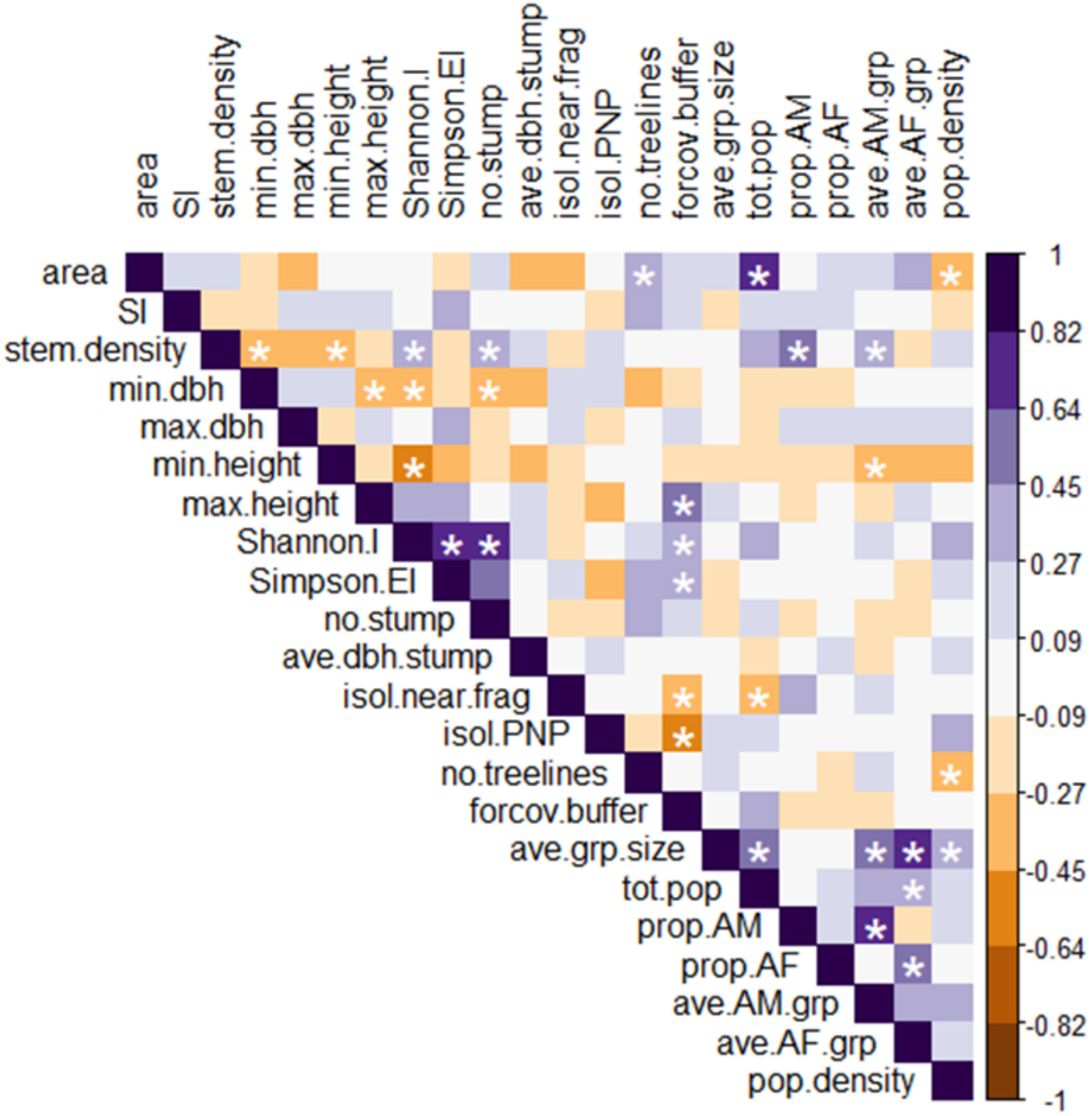
Heatmap of Spearman rank correlations among all explanatory and response variables. Scale on the right denotes magnitude and direction of correlations among pairs of variables; white asterisks denote statistically significant correlations (*p* < 0.05). Definitions of abbreviated variable names: *explanatory variables*: area, fragment area; SI, shape index; stem.density, stem density; min.dbh, min. tree DBH; max.dbh, max. tree DBH; min.height, min. tree height; max.height, max. tree height; Shannon.I, Shannon’s index; Simpson.EI, Simpson’s evenness index; no.stump, number of stumps; ave.dbh.stump, mean DBH of stumps; isol.near.frag, distance to nearest fragment; isol.PNP, distance to PNP; no.treelines, number of treelines extending from fragment; forcov.buffer, proportion of forest cover in the buffer. *Response variables*: ave.grp.size, mean group size in fragment; tot.pop, total number of individuals per fragment; prop.AM, proportion of AM in the fragment population; prop.AF, proportion of AF in the fragment population; ave.AM.grp, mean number of AM per group in the fragment; ave.AF.grp, mean number of AF per group in the fragment; pop.density, fragment population density.

The Spearman correlation heatmap (Fig. 4; see Table SM3 for all correlation coefficients and *p*-values) showed that mean group size was significantly positively correlated with four other response variables: population size, population density, the mean #AM/grp, and the mean #AF/grp. Given this redundancy, we excluded mean group size from the RDA analysis.

**Figure 5:**
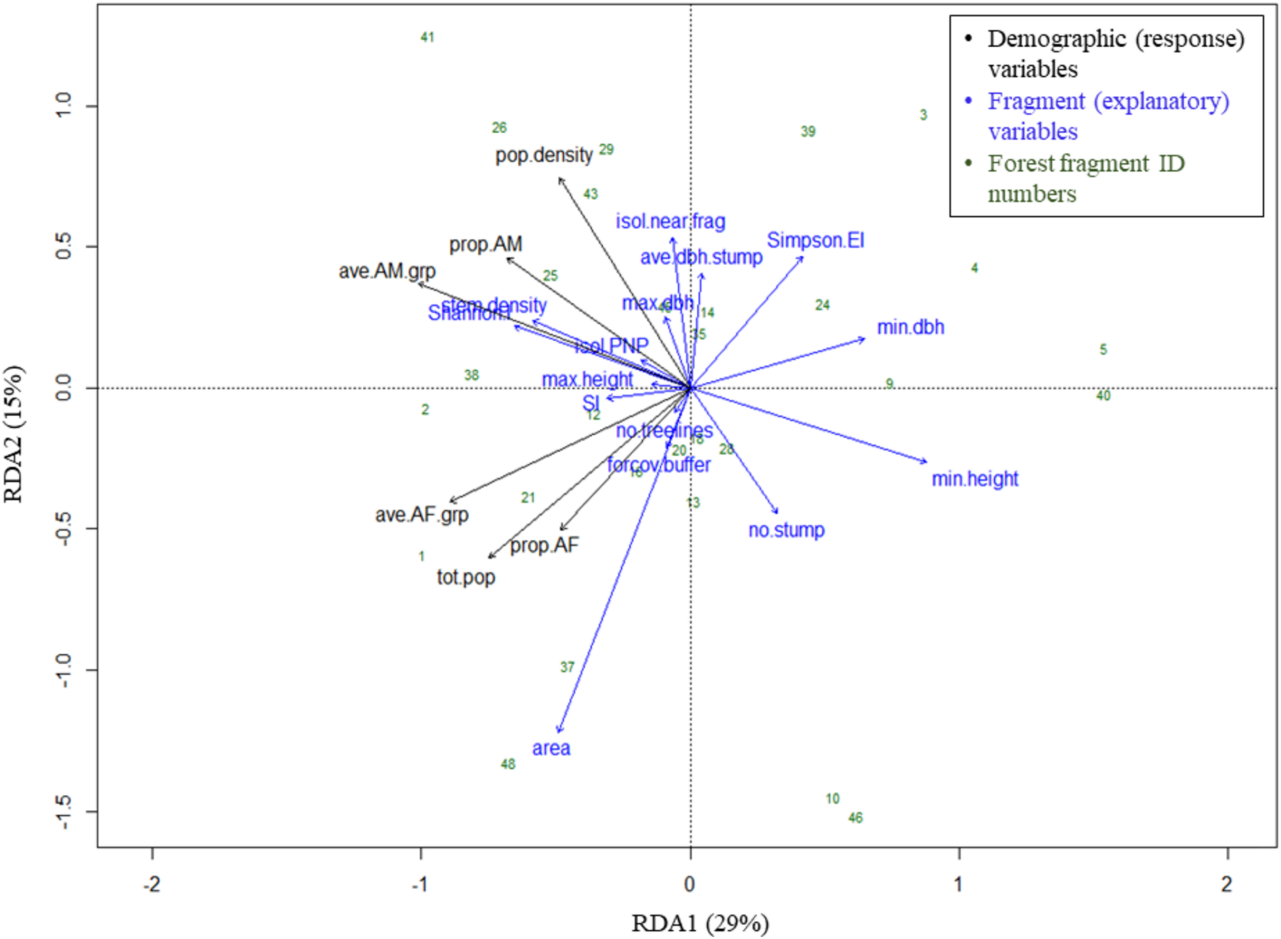
Triplot of RDA results: effects of fragment variables on black howler monkey population size, density and composition in forest fragments. Proportion of variance in demographic data explained by each axis noted in parentheses in axis labels. For definitions of variable names, see Fig. 4.

The RDA model with 15 fragment variables accounted for over two-thirds of the variation in the demography of the black howler population in forest fragments (*R*^2^ = 0.69). This model was globally significant (*p* = 0.003, *F* = 1.967, df = 15), and RDA axis 1 was also independently significant (*p* = 0.018, *F* = 20.93, df = 1), although axis 2 was not (*p* = 0.223, *F* = 10.63, df = 1; Fig. 5). Of the individual explanatory variables, the marginal effects of three were statistically significant (fragment area, Simpson’s evenness index, and the number of stumps) and two were close to the significance value (stem density and Shannon’s index; Table 3). The variables most strongly driving axes 1 and 2, which together accounted for 44% of the explained variation in the demographic variables (Fig. 5), were (in descending order) minimum tree height, Shannon’s index, minimum tree DBH, stem density, fragment area, and Simpson’s evenness index (axis 1); and area, the isolation distance to the nearest fragment, Simpson’s evenness index, the number of stumps, and the mean DBH of stumps (axis 2; Table 4).

**Table 3:** Marginal effects of individual explanatory variables in the RDA model.

Variables	Variance	*F*-statistic	*p*-Value
Area (ha)^a^	0.35	2.50	0.03
Shape index	0.14	1.02	0.41
Stem density	0.31	2.18	0.07
Min. tree DBH (cm)	0.17	1.19	0.30
Max. tree DBH (cm)	0.16	1.11	0.39
Min. tree height (m)	0.13	0.95	0.46
Max. tree height (m)	0.14	1.02	0.42
Number of stumps^a^	0.45	3.16	0.02
Mean DBH of stumps (cm)	0.05	0.32	0.89
Shannon’s index (genera)	0.31	2.23	0.07
Simpson’s evenness index (genera)^a^	0.38	2.73	0.03
Isolation distance to nearest fragment (m)	0.07	0.53	0.75
Isolation distance to PNP (m)	0.05	0.37	0.85
Number of tree lines extending from fragment	0.19	1.37	0.26
Proportion forest cover in buffer	0.12	0.88	0.47

**Note:**

a Significant at α = 0.05.

**Table 4:** Triplot scores for constraining (explanatory) variables and response variables.

	^c^Axis 1 (29%)	^c^Axis 2 (15%)
**^a^Explanatory variables**		
Area (ha)	−0.30	−0.75
Shape index	−0.19	−0.02
Stem density	−0.36	0.15
Min. tree DBH (cm)	0.40	0.11
Max. tree DBH (cm)	−0.06	0.15
Min. tree height (m)	0.54	−0.16
Max. tree height (m)	−0.09	0.01
Number of stumps	0.20	−0.27
Mean DBH of stumps (cm)	0.03	0.25
Shannon’s index (genera)	−0.40	0.13
Simpson’s evenness index (genera)	0.25	0.29
Isolation distance to nearest fragment (m)	−0.04	0.33
Isolation distance to PNP (m)	−0.11	0.06
Number of tree lines extending from fragment	−0.04	−0.05
Proportion forest cover in buffer	−0.05	−0.13
**^b^Response variables**		
Total number of individuals per fragment	−0.83	−0.67
Fragment population density	−0.54	0.83
Proportion of AM in the fragment population	−0.76	0.51
Proportion of AF in the fragment population	−0.54	−0.56
Mean #AM per group in the fragment	−1.13	0.41
Mean #AF per group in the fragment	−0.99	−0.45

**Notes:**

a These scores are the coordinates of the vector tips for each explanatory variable. These coordinates are obtained by computing correlations between the explanatory variables and the fitted site scores (i.e., the scores for each fragment; Borcard, Gillet & Legendre, 2018). The magnitude of the score indicates the magnitude of the variable’s contribution to the axis, and the sign (+/−) indicates the direction of variable’s vector along the axis. For example, the isolation distance to the nearest fragment had a relatively large contribution to axis 2, and correlated positively with axis 2, and thus with the response variables that correlated positively with axis 2, such as population density.

b These scores are the coordinates of the vector tips for each response variable (Borcard, Gillet & Legendre, 2018).

c In parentheses: the percentage of the variation in response variables explained by each axis.

Geographically close fragments did not cluster together in the triplot, indicating that proximity in the landscape did not predict similarity in black howler demography (Figs. 3 and 5). Similarly, those fragments located in the more connected southern area around PNP did not cluster together in the triplot; aside from the Shannon’s and Simpson’s indices, the main variables that drove the variation in fragment demography were not those that differed among the more vs. less connected fragments.

The proportion of AF and average #AF/grp variables clustered together (and with the fragment population size), correlating negatively with both axes (Fig. 5; Table 4). These response variables were most closely positively associated with fragment area, the number of treelines extending from the fragment, Shannon’s index, stem density, the proportion of forest cover in the buffer, and the shape index, although area had the strongest effect. These variables showed the strongest negative correlations with Simpson’s evenness, the minimum tree DBH and height, and the mean stump DBH (Fig. 5; Table 4).

Variables related to adult males also grouped together (and with population density), correlating negatively with axis 1 and positively with axis 2 (Fig. 5; Table 4). These variables were most closely positively associated with stem density, the distance from PNP, the Shannon index, maximum tree DBH, mean stump DBH, and the isolation distance to the nearest fragment (particularly density and the proportion of AM in the fragment; Fig. 5; Table 4); of these, Shannon’s index, stem density, and isolation from the nearest fragment had the strongest effect. The AM variables were negatively correlated with minimum tree height and DBH and the number of stumps. Population density was negatively correlated with area, the proportion of forest cover in the buffer, minimum tree height, and the number of stumps (Fig. 5; Table 4).

Several significant pairwise relationships emerged from the Spearman correlation heatmap (Fig. 4) that further clarify the patterns found in the RDA analysis. Some discrepancies in the heatmap and RDA results are expected, as RDA performs a joint analysis of the two datasets (i.e., demographic and fragment variables) to assesses the degree of co-variation between them; in other words, RDA identifies patterns that occur in both datasets, and ignores patterns that are unique to only one (Ulhaq et al., 2013).

Among the six response variables, only the mean #AF/grp was significantly and positively correlated with population size. Among the explanatory variables, the proportion of forest cover in the buffer was significantly positively correlated with maximum tree height and Simpson’s evenness index, and negatively correlated with the isolation distance from PNP. These relationships further supported the results of the analysis comparing the more connected fragments to the less connected ones, as defined by FRAGSTATS. Stem density was significantly negatively correlated with minimum tree height and DBH and positively correlated with Shannon’s index, and also (unlike the relationship seen in the RDA) positively correlated with the number of stumps. Similarly, the strong positive correlation between the Shannon’s and Simpson’s evenness indices seen in the heatmap was not apparent in the RDA.

The heatmap showed several significant relationships among individual explanatory and response variables. Fragment area correlated positively with population size and negatively with population density. Density also correlated negatively with the number of treelines extending from the fragment, and population size correlated negatively with the isolation distance from the nearest fragment. The proportion of AM and mean #AM/grp both correlated positively with stem density, while the mean #AM/grp also correlated negatively with minimum tree height.

## Discussion

We examined the effects of variation in forest fragment habitat and connectivity on the within-fragment size, density, and demography of populations of black howlers living in unprotected forest fragments around PNP, Chiapas, Mexico.

It is important to note that fragment and landscape variables not included in our analyses may also play an important role in shaping population demography, via effects on movement through the landscape and fragment carrying capacity. For example, the different matrix types surrounding fragments, especially given the heterogeneous nature of the matrix in the study landscape (Eycott et al., 2012), and in particular the presence of large oil palm plantations near some fragments, which may severely limit movement (Mendes-Oliveira et al., 2017). Population demography in fragments in the present may also be shaped by past events (e.g., hunting, natural disasters; Pavelka et al., 2003), altered patterns of disease prevalence, transmission and mortality in fragments (Gottdenker et al., 2014), or demographic stochasticity, which is more common in the small population sizes found in forest fragments (Lawler, 2011).

However, our results clearly showed that forest loss and fragmentation are ongoing threats in our study site that are also altering conditions within fragments, and that variation in fragment characteristics has played a substantial role in shaping black howler demography in forest fragments, with differential effects on adult males and females. These results have implications for our understanding of how black howlers, and other similar, arboreal species of primates inhabiting fragmented tropical forest landscapes, respond to forest loss and fragmentation, and for the population’s long-term persistence in the area.

Hypothesis 1: characteristics of forest fragments and landscape connectivity in the vicinity of fragments have a measurable, significant effect on the population size and density of black howlers living in fragments

The RDA results provided clear support for the hypothesis that fragment variables play a substantial part in shaping black howler population size and density in fragments around PNP: population size in fragments increased with fragment area and connectivity in the vicinity of the fragment, while population density decreased with increased area and local connectivity. Indeed, area proved to have a very strong effect on population demography in fragments, particularly population size, having a significant marginal effect in the RDA model and contributing substantially to both RDA axes.

These results track with the Pardini et al. (2010) study that showed that patch area had the strongest effect on forest specialist abundance in landscapes with intermediate amounts of habitat cover, such as our study area (38%). Similar area effects have been found for other arboreal primates (Umapathy & Kumar, 2003; Anzurres-Dadda & Manson, 2007; Irwin, 2008; Arroyo-Rodriguez & Dias, 2010). It is interesting to note that the increase in population size with fragment area in our study was driven entirely by an increase in AF (and presumably, their offspring). While Puig-Lagunes et al. (2016) found that numbers of all age-sex classes increased with fragment size and connectivity in mantled howlers in fragments, when Mandujano & Escobedo-Morales (2008) modeled population viability for mantled howlers under landscape scenarios of varying connectivity, they also found that reproductive rates and population growth rates in fragments depended mainly on the number and survival of adult females.

Arroyo-Rodriguez et al. (2013b) found that black howler abundance was positively associated with higher landscape connectivity, and our results also showed that population size in fragments increased with greater connectivity in the vicinity of fragments: population size negatively correlated with isolation distance to the nearest fragment and correlated positively with the proportion of forest cover in the buffer. Connectivity at the broader landscape scale, as identified in the FRAGSTATS analysis, appeared to have a lesser impact on black howler demography within individual fragments. Arroyo-Rodriguez et al. (2013b) similarly found that black howler monkey demography was more affected by local-scale metrics.

Not all studies of species in fragmented landscapes, or even arboreal primates specifically (DeGama-Blanchet & Fedigan, 2006) find that density increases with decreasing fragment size. In their review of fragment area-density relationships in mammals, Bowers & Matter (1997) found that area does not have a linear effect on density, but rather that negative density-area relationships are more common in landscapes with smaller fragments; our study supports this finding (range of fragment sizes: 0.2–36.2 ha). Bowers & Matter (1997) also found that landscapes with less isolated fragments tended to have this negative area-density relationship; in contrast, both our study and Arroyo-Rodriguez et al. (2013b) found that higher density was correlated with more isolated fragments for black howlers.

Hypothesis 2: variation in the composition of black howler populations in fragments is shaped by differential effects of fragment characteristics and fragment connectivity on adult males and females

Our results supported the hypothesis that adult males and females are differentially affected by the fragmented landscape, clearly grouping by sex in the RDA triplot. Adult males and females differed in their responses to the fragment variables that contributed most substantially to axis 2, namely area, the isolation distance to the nearest fragment, Simpson’s evenness index, the number of stumps, and the mean DBH of stumps. Our hypotheses regarding the variables affecting each sex were partially supported by our results.

### Adult females

Based on additional analyses of this population (Klass, Van Belle & Estrada, 2020) and of black-and-gold howler monkeys (Oklander & Corach, 2013) indicating that howler monkey females may be more philopatric in fragmented landscapes, we hypothesized that in our study fragments, AF become more philopatric than in continuous forests because of increased competition for resources (Gros, Poethke & Hovestadt, 2009). We therefore expected variables relating to AF to positively correlate with variables indicative of habitat quality, where we defined habitat quality as increasing with increasing similarity to primary, undisturbed rainforest.

The main patterns that emerged for AF can be summarized thus: there were more AF/group and a higher proportion of AF in the population in larger fragments with better connectivity, characteristics indicative of abundant secondary growth (e.g., high stem density, smaller minimum tree size), and those with more diverse vegetation, but lower Simpson’s evenness indices (indicative of a tree composition dominated by only one or a few genera; Morris et al., 2014). Adult females were also more abundant in fragments with smaller mean stump size, which may be an indication of less human disturbance, as larger stumps were often the result of the harvesting of large, mature trees by local people.

These results are somewhat surprising, not least because the RDA shows that while there were more AF in larger fragments and in fragments with more diverse tree genera, there was no direct correlation between area and tree diversity, and a negative correlation between area and the evenness of the forest composition; we would expect larger fragments, with fewer edge effects, to have more diverse vegetation, with a more even Simpson’s index (Santos et al., 2008; Dias et al., 2014). However, fragment size does not always positively correlate with food availability (Dias et al., 2014), and it is possible that the ability of black howlers to maintain dietary breadth in fragments with different levels of tree species richness and diversity (Dias et al., 2014) minimizes the importance of tree composition for black howler females. Productive secondary growth in fragments may provide howlers with abundant food resources (Arroyo-Rodriguez & Dias, 2010), albeit different from those found in undisturbed primary rainforest.

Another indication that secondary growth food resources may be important for howlers in fragments is the positive, although minor, association we found between the shape index and many of the response variables. A high shape index indicates a complex fragment shape with more forest edge, which is associated with changes to vegetation relative to primary forest that could be considered indicative of low-quality habitat with fewer resources (Ewers & Didham, 2007; Arroyo-Rodriguez & Dias, 2010). However, fragments with more complex shapes and more edges may also have an abundance of new, secondary growth, providing howlers with an important source of young leaves to feed on (Cristobal-Azkarate et al., 2005).

We found that fragment isolation negatively affected the abundance of AF, which may stem from females avoiding costly, long-distance dispersal through the matrix. Additional studies have shown that habitat fragmentation may reduce female dispersal frequency (Oklander & Corach, 2013) or distance traveled (Minhos Rodrigues, 2012) in arboreal primates.

In continuous forest populations of ursine howler monkeys (*A. arctoidea*), the costs of dispersal in terms of reproductive success and mortality risks were much greater for females than for males, and females would thus prefer to remain, and have their daughters remain, in the natal group and territory when conditions allowed (e.g., <4 AF in the group; Pope, 2000). When obligated to disperse, females encountered resistance to immigration into existing groups from resident females, thereby increasing dispersal costs. Evidence from PNP indicates that female black howler dispersal patterns in continuous forests may be similar to ursine howlers (Van Belle et al., 2012; S. Van Belle, 2019, unpublished data). Thus, a possible explanation for the patterns found in this study is that black howler females in fragments are more likely to remain in the natal group, establish a new group in the natal fragment, or disperse short distances to nearby fragments, rather than disperse long distances across the matrix. This is because the benefits of remaining in the natal group or fragment, in terms of access to resources and reproductive success, are even greater than in continuous forests, as the potential costs of dispersal outside of the natal fragment, in terms of mortality risk and energetic costs, are likely even higher in a fragmented landscape compared to continuous forest (Bowler & Benton, 2005).

Avoidance of longer-distance dispersal by females may only be possible for those in larger, less-isolated fragments, that can support multiple groups or are near easily accessible additional fragments; thus over time populations in these fragments might show higher proportions of AF and larger populations overall, as we found in this study. This strategy may not be available to females born in smaller, more isolated fragments, as establishing new groups in these small areas would be difficult; additionally, our results showed that populations in smaller, isolated fragments have fewer AF, not more.

Similarities between the study fragment population and populations in PNP and other continuous forests in group size and mean number of AF (Klass, Van Belle & Estrada, 2020) may indicate that, when possible, females in fragments may be more commonly avoiding high dispersal costs by establishing new groups in the natal fragment or dispersing to nearby fragments, rather than remaining in the natal group more often than in continuous forest populations. The high per-fragment densities in our study fragments may provide some support for this scenario; these densities were often the result of multiple groups simultaneously occupying fragments smaller than some black howler group home range sizes observed in continuous forests (Gavazzi et al., 2008; De Guinea et al., 2019).

### Adult males

Based on additional analyses of this population (Klass, Van Belle & Estrada, 2020) and on the hypothesis that males continue to disperse in the fragmented landscape to seek reproductive opportunities (Gros, Poethke & Hovestadt, 2009) and suffer higher mortality during dispersal (Banks et al., 2005), we predicted that the proportion of AM in the fragment and mean #AM/group in forest fragments would correlate positively with variables quantifying connectivity around fragments.

The main patterns that emerged for AM can be summarized thus: there were more AM/group and a higher proportion of AM in the population in fragments that were more isolated from nearby fragments (and to a lesser extent, from PNP), with characteristics indicative of abundant secondary tree growth (e.g., high stem density, smaller minimum tree size) but larger maximum tree DBH, with fewer, larger stumps, and more diverse vegetation, but lower Simpson’s evenness indices. Thus, contrary to our prediction, we found AM were more abundant in more isolated fragments. The abundance of AM was uncorrelated to fragment area, in contrast both to Dias et al. (2015) study on black howlers in fragments in Campeche, Mexico, in which groups were found to include more males in smaller fragments, and Puig-Lagunes et al. (2016), who found that numbers of all age-sex classes increased with fragment size in mantled howlers.

Pope (2000) found that ursine howler males in continuous forests most often dispersed very short distances, to neighboring groups, and when attempts to join or take over those groups were unsuccessful, they returned to the natal group, thus incurring generally low dispersal costs. Evidence from PNP indicates that black howler male dispersal patterns in continuous forests may be similar (S. Van Belle, 2019, unpublished data). However, AM in forest fragments are faced with a scarcity of nearby, familiar groups to take over or join, and as a result may be dispersing at similar rates but forced to travel longer distances during dispersal in the fragmented landscape as compared to continuous forest, to seek reproductive opportunities in more isolated fragments.

While engaged in longer-distance dispersal through the matrix, black howler males may be subject to increased mortality risks. Pozo-Montuy, Serio-Silva & Bonilla-Sanchez (2011) showed that while overall rates of emigration may be similar in continuous and fragmented habitat for black howlers in Balancán, Mexico, increased mortality for dispersers moving through the matrix in fragmented landscapes may result in reduced effective dispersal rates and gene flow. Hunting of black howlers occurs in our study area, and dogs are known to kill monkeys forced to move on the ground (Pozo-Montuy, Serio-Silva & Bonilla-Sanchez, 2011), which occurs in the matrix in our study site (Fig. 2D). The occurrence of increased male mortality in the fragmented landscape is supported by additional analyses of this population, which indicated that there were fewer AM in forest fragments relative to the population in PNP (Klass, Van Belle & Estrada, 2020).

The minimal effect of distance from PNP on the demography of fragment populations indicated that the mainland-island metapopulation model (Hanski, 1998) does not apply to the study population and the nearby large, stable population in PNP; that is, PNP does not act as a significant source population for the fragments (source-sink dynamics; Pulliam, 1988), including those that are closer to the park. However, it is possible that the larger, more productive fragments in the landscape, which are able to support multiple groups, may act as a source for small, sink populations in the more isolated, lower-quality fragments (Pulliam, 1988; Arroyo-Rodriguez, Mandujano & Benitez-Malvido, 2008). According to Banks et al. (2005), while high male dispersal mortality may bias the overall population sex ratio towards females, small populations maintained by immigration (i.e., sink populations) may have a relatively higher proportion of the dispersing sex. This pattern is in line with our observations of the study population: both fewer AM overall compared to PNP (Klass, Van Belle & Estrada, 2020) and higher proportions of AM and more AM/group in more isolated fragments.

## Conclusions: Implications for Conservation

Fragment area explained much of the variation in black howler population demography, particularly for adult females and population size. In terms of persistence in the fragmented landscape, this may be encouraging, in that black howlers seem adaptable to considerable variation in habitat composition and quality, at least in the short term. However, while the population in fragments has been stable for ~20 years (Klass, Van Belle & Estrada, 2020) it is unclear whether it can persist over longer timescales, and how increasing fragment isolation and changing forest compositions may interact with fragment area to affect individual fitness and long-term population persistence (extinction debt; Tilman et al., 1994; Fletcher et al., 2018). Additionally, the fact that a high rate of deforestation is ongoing in the study area is alarming; even the extremely minimal requirement of an area of forest is becoming scarcer in the landscape around PNP. Given the small size of most fragments in this area, it is likely that most, if not all, cannot sustain viable populations (Ewers & Didham, 2006; Laurance et al., 2017); rather, black howlers may only be able to persist in this landscape as a functional metapopulation (Hanski, 1998; Fahrig & Merriam, 1994), for which successful dispersal between fragments is critical (Dale, 2001; Ewers & Didham, 2006; Baguette et al., 2012). The importance of dispersal for maintaining spatially fragmented populations highlights the significance of our findings regarding differential effects of fragment characteristics on males and females: if females are indeed remaining in their natal groups or fragments more, while males tend to disperse longer distances across the matrix to more isolated fragments, it would indicate that to effectively conserve this population over time, both landscape connectivity and habitat area need to be maintained and increased.

## Supplemental Information

10.7717/peerj.9694/supp-1 Supplemental Information 1 Detailed fragment and demographic data for all fragments.

10.7717/peerj.9694/supp-2 Supplemental Information 2 List of all tree families and genera sampled in vegetation transects in the study fragments.

10.7717/peerj.9694/supp-3 Supplemental Information 3 Spearman correlation values of pair-wise correlations among all explanatory and response variables. *p*-Values denoted as follows: 0.1 > *p* > 0.05; *p < 0.05; **p <0.01; ***p < 0.001; ****p < 0.0001.

## Additional Information and Declarations

### Field Study Permissions

The following information was supplied relating to field study approvals (i.e., approving body and any reference numbers): Data collection was conducted under a research permit from Dirección General de Vida Silvestre de la SEMARNAT (SGPA/DGVS/05700/17; SGPA/DGVS/002486/18).

### Data Availability

The following information was supplied regarding data availability: The raw data used in our analyses are available as Supplemental Files.
